# Pan-genome insights into genetic diversity, evolutionary dynamics, and pathogenic traits of *Staphylococcus agnetis*

**DOI:** 10.1186/s12864-026-13008-y

**Published:** 2026-06-03

**Authors:** Meng Wang, Jingyu Wang, Ce Wang, Congcong Liu, Jie Chen, Yue Liang, Jing Liu, Caihong Yang, Zhiqiu Yin, Chunlei Zhou, Hong Mu, Yuhui Du

**Affiliations:** 1 Department of Clinical Laboratory, School of Medicine, Tianjin First Central Hospital, Nankai University, Tianjin, 300457 PR China; 2 Department of Clinical Laboratory, Key Laboratory of Biological Targeting Diagnosis, Therapy and Rehabilitation of Guangdong Higher Education Institutes, The Fifth Affiliated Hospital, Guangzhou Medical University, Guangzhou, Guangdong 510700 China; 3 Engineering Technology Research Center of Intelligent Diagnosis for Infectious Diseases in Guangdong Province, Guangzhou, 511436 China; 4 School of Biology and Biological Engineering, South China University of Technology, Guangzhou, Guangdong 510006 China

**Keywords:** *Staphylococcus agnetis*, Pan-genome, Evolutionary dynamics, Gene gain, Gene loss, Virulence factor, Antimicrobial resistance

## Abstract

**Background Staphylococcus agnetis:**

is an emerging pathogen primarily associated with bovine mastitis and avian lameness. Despite increasing reports of its occurrence across animal hosts, its genomic diversity and the distribution of antimicrobial resistance (AMR) and virulence-associated genes remain insufficiently characterized.

**Results:**

The species *S. agnetis* possesses an open pan-genome, dominated by cloud gene families enriched in defense mechanisms and genomic plasticity, consistent with gene flux. Evolutionary reconstruction indicated that purifying selection and gene loss are the main signatures of evolutionary dynamics in the *S. agnetis* pan-genome, with extensive gene loss particularly affecting cell wall biogenesis functions. Notably, significant gene gain events were observed at early-diverging internal nodes of the phylogeny, suggesting that gene acquisition occurred during the early diversification of *S. agnetis*. AMR profiling identified a limited repertoire of AMR genes. However, the detection of a plasmid-borne AMR gene and the distribution of plasmids highlight the potential for plasmid-mediated dissemination of AMR in *S. agnetis*. Virulence profiling identified 28 chromosomally located putative virulence-related genes, predominantly homologous to *S. aureus*, including core adherence factors and sporadically distributed enterotoxin homologs suggestive of acquisition via horizontal gene transfer (HGT).

**Conclusions:**

Collectively, this study provides comprehensive insights into the genomic diversification of *S. agnetis* and highlights its emerging AMR traits and putative virulence potential in animal-associated settings.

**Supplementary Information:**

The online version contains supplementary material available at 10.1186/s12864-026-13008-y.

## Background

*Staphylococcus* is a genus of Gram-positive, non-motile, facultatively anaerobic cocci, currently comprising more than 70 species with validly published names according to the List of Prokaryotic names with Standing in Nomenclature (LPSN) [1, 2]. This genus includes saprophytic species in diverse environments [3–5] and many species that are commensals of diverse hosts including mammals and birds [6–9]. Certain species can become opportunistic pathogens by secreting enzymes, such as hyaluronidases and proteases, that degrade epithelial barriers, breach the structural integrity of these barriers, and thereby facilitate the establishment of infection [10]. In addition, staphylococcal pathogenesis is multifactorial, involving adhesion mediated by microbial surface components recognizing adhesive matrix molecules, the production of cytolytic toxins that damage host cells, and biofilm formation that facilitates persistence, along with the capacity for systemic dissemination [1, 11, 12]. Immunosuppressive states induced by physiological stress or immune dysregulation can compromise host defenses, thereby increasing susceptibility to staphylococcal colonization and infection [13]. Compared to the well-characterized pathogen *Staphylococcus aureus*, several species within this genus, including *Staphylococcus agnetis*, remain relatively understudied, particularly with respect to their genomic diversity, antimicrobial resistance profiles, and virulence-associated features. Therefore, combined phylogenetic, antimicrobial resistance (AMR), and virulence profiling using comparative and pan-genome genomics is needed to better define the diversity and potential pathogenic features of emerging staphylococcal species and to inform surveillance and control efforts.

*S. agnetis* was first recognized as a distinct species from cases of subclinical bovine mastitis in 2012 [14, 15]. It exhibits coagulase-variable phenotypes that are characteristic of staphylococci that are prevalent in bovine mammary infections [14, 16]. In addition to causing mastitis, this pathogen induces avian lameness through chondrolytic destruction and concurrent osteomyelitis [17, 18]. This was validated experimentally by Alrubaye et al., who demonstrated its capacity to trigger bacterial chondronecrosis with osteomyelitis in broiler chickens via contaminated drinking water. This indicates that waterborne exposure may contribute to transmission under experimental conditions, although the natural transmission routes in field settings remain to be fully elucidated [18, 19]. Phylogenetic analyses of *S. agnetis* isolates from cattle and chickens revealed a close genetic relationship, suggesting a recent host-jump event and indicating its capacity for cross-species infection [20]. *S. agnetis* harbors conserved virulence factors that may contribute to cross-species pathogenicity [21]. Biofilm formation, often associated with the *icaADBC* operon, contributes to persistent colonization of epithelial surfaces and milking equipment in bovine mastitis [22]. The detection of staphylococcal enterotoxin (SE) genes (e.g., *seh*, *sej*, and *sep*) in a subset of poultry-derived isolates suggests that these strains may serve as potential reservoirs of enterotoxin genes [23]. Furthermore, the horizontal transfer of chromosomal islands mediating cross-species virulence adaptation has been reported between *S. aureus* and *S. agnetis* [21]. *S. agnetis* is also recognized as a potential reservoir of AMR genes, including β-lactam resistance determinants such as *blaZ*, as well as *femA*/*femB* and macrolide (*erm* classes) and lincosamide (*lnu*(A)) resistance genes [24]. Therefore, a more detailed explanation of the genetic diversity, underlying pathogenicity and resistance mechanisms of this bacterium is required.

Pan-genome analysis is a powerful method for understanding the genetic potential of a bacterial species [25, 26]. In this study, we provide a comprehensive genomic perspective on *S. agnetis*, integrating phylogenomic analysis, pan-genome characterization, and comparative genomic approaches to investigate its genetic diversity, evolutionary dynamics, and the distribution of virulence and AMR determinants.

## Methods

### Genome collection and filtering

A comprehensive search for *S. agnetis* genomes was conducted using EzBioCloud [27] and NCBI GenBank. All *S. agnetis* genomes were downloaded from the NCBI GenBank database on 17 August 2024, yielding 62 genomes in total. The quality assessment of these genomes was conducted via CheckM v1.0.13 [28]. We excluded genomes with an excessive number of contigs (> 300), less than 95.0% completeness, or more than 10.0% contamination. Two genomes (GCA_003040895.1 and GCA_012029365.1) were removed due to excessive contig numbers (> 300). In addition, all genomes were compared with the reference genome (strain 1379: GCA_011466855.1) using fastANI v2.0 [29], and the genomes that exhibited < 95.0% average nucleotide identities (ANI) values to the reference were excluded. Following these stringent filters, our final collection consisted of 60 *S. agnetis* genomes with high assembly quality (mean contig number = 55.2 ± 46.3; completeness = 99.3% ± 0.3%; contamination = 1.1% ± 1.0%) (Table S1), complemented by one reference genome of closely related species (*Staphylococcus hyicus* ATCC 11249: GCA_000816085.1). Variation in assembly fragmentation was taken into account during downstream pan-genome analyses, particularly for gene presence/absence inference, pan-genome partitioning, and the identification of putative virulence and AMR genes. Fragmented assemblies can lead to missing or truncated gene predictions, which may affect estimates of core and accessory gene content [30, 31]. To reduce this bias, genomes were filtered using stringent quality criteria (≥ 95% completeness, ≤ 10% contamination, and contig number ≤ 300), and only high-quality assemblies were retained.

### Pan-genome and phylogenomic analysis

To ensure unified gene finding and reannotation, we utilized Prokka v1.14.5 software [32] for all the genomes in our collection. The resulting GFF files were analyzed using Panaroo v1.3.4 with the default settings to determine the core gene alignment [33], which was used to construct the maximum-likelihood (ML) tree using IQ-TREE v2.2.5 with the general time reversible model and 100 bootstrap replicates [34]. The Panaroo output files were used to extract pan-gene families (the totality of all genes found across strains), core gene families (genes shared by ≥ 99.0% of genomes), soft core gene families (genes shared by ≥ 95.0% of genomes and < 99.0% of genomes), shell gene families (genes shared by ≥ 15.0% of genomes and ≤ 95.0% of genomes), and cloud gene families (genes shared by ≤ 15.0% of genomes). For core-genome analysis of *S. agnetis*, 60 genomes were aligned using Parsnp v2.1.1 [35], with *S. agnetis* 1379 (GCA_900475375.1) used as the reference genome. A ML phylogenetic tree was constructed from core-genome single-nucleotide polymorphisms (SNPs) identified by Parsnp v2.1.1 using FastTree v2.1.10 [36]. Pyani v0.3.0-alpha with the MUMmer model (ANIm) was used to calculate the ANI values [37, 38]. The functional annotation of pan-genome gene families was performed based on the Clusters of Orthologous Groups (COG) categories [39] using eggNOG-mapper 2.1.9 software [40]. Functional enrichment of COG categories among different components of the *S. agnetis* pan-genome was assessed using Fisher’s exact test.

### Population structure analysis

The population genetic structure of *S. agnetis* was inferred via STRUCTURE 2.3.4 [41] based on the core gene alignment, employing 20,000 burn-in cycles, 20,000 sampling Markov chain Monte Carlo cycles, *K* values ranging from 2 to 12, and five independent replicates. The optimal *K* value, indicative of the most likely number of genetic clusters, was identified via StructureSelector [42].

### Gene gain and loss inference

To provide insight into the evolutionary dynamics of gene families in the pan-genome, we inferred ancestral family sizes and the patterns of gene gain and loss at each node/tip of the core genome tree via the COUNT program with the Dollo parsimony model [43], which strictly prohibits multiple gains of genes and allows the reconstruction of gene gain and loss events in both observed species and potential ancestors (nodes/tips on the phylogenetic tree) [44].

### Selective pressure analysis

Codon-level ratios of nonsynonymous to synonymous substitution rates (*dN*/*dS*) were calculated for 3284 pan-gene families (present in ≥ 4 genomes). ParaAT 2.0 was used for codon-based alignment of orthologous genes [45]. The fast unconstrained Bayesian approximation (FUBAR) pipeline [46] in HYPHY v2.5.42 was used to measure *dN*/*dS* ratio at each site in orthologous gene families. Codon sites under diversifying positive selection were inferred at a posterior probability ≥ 0.9.

### Comparative genomic analysis

Prediction of AMR phenotypes and detection of AMR genes were carried out using ResFinder v4.3.1 [47]. In order to identify mobile genetic element (MGE)-driven AMR genes, the VRprofile2 was utilized, which incorporates SIGI-HMM and IslandPath-DIMOB with other computational methods in a parallel workflow to identify integrons, prophages, integrative and conjugative elements (ICEs), plasmids, transposons, and known AMR genes [48]. The Deeplasmid tool was used to distinguish plasmid sequences from assembled contigs or scaffolds [49]. Putative virulence-related genes were detected using Abricate v1.0.1 (https://github.com/tseemann/abricate) with the Virulence Factors Database (VFDB) [50], with a cutoff at 60% nucleotide identity and 60% nucleotide coverage. This relatively permissive threshold was applied because VFDB does not contain curated virulence factors for *S. agnetis*, and detection therefore relies on identifying homologs to virulence genes characterized in related species (e.g., *S. aureus*). Similar thresholds have been adopted in previous studies investigating underrepresented or non-model bacterial species [51]. Consequently, the identified genes should be interpreted as putative virulence-related homologs rather than confirmed virulence determinants.

### Visualization of genomic data and statistical plots

The circular representation of the reference genome was generated using the BLAST Ring Image Generator (BRIG) [52]. Heatmaps of ANIs were produced in R using the pheatmap v1.0.13 package. Phylogenetic trees were visualized and edited using FigTree v1.4.4 (http://tree.bio.ed.ac.uk/software/figtree/).

The expanded view of the MGE region was generated using VRprofile2 with default annotations. Statistical plots were generated using a combination of tools. Box plots were created in Microsoft Excel. Violin plots were generated using R (ggplot2 v0.9.3.1 package). Alluvial plots were constructed using the RAWGraphs 2.0 web application (https://app.rawgraphs.io/).

## Results and discussion

### The genomic landscape of *S. agnetis*

**Fig. 1:**
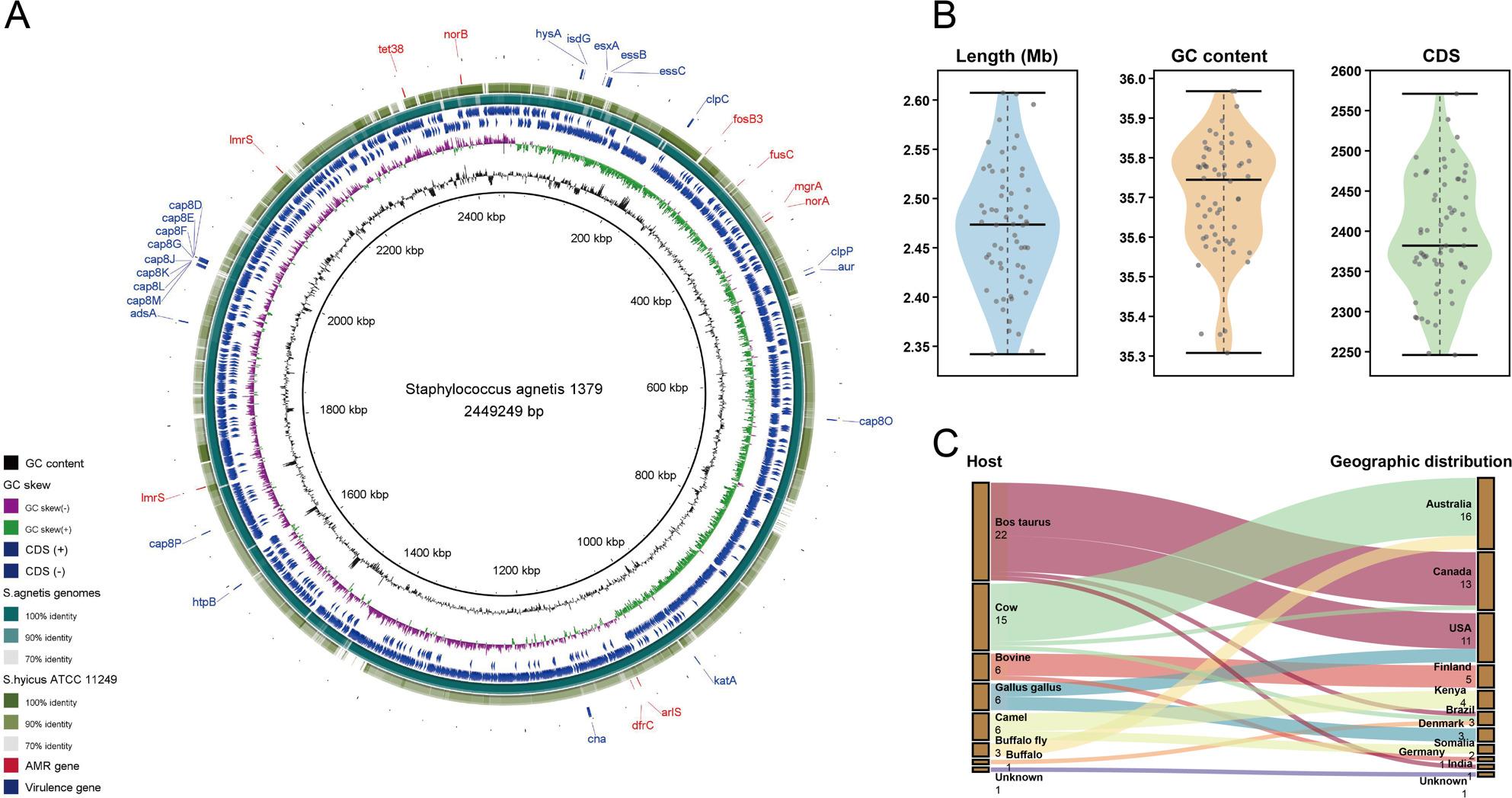
**A** Circular representation of the S. agnetis reference genome (strain 1379: GCA_011466855.1). **B** General genomic characteristics of 60 S. agnetis genomes. The violin plots illustrate the distribution of genome sizes, GC content, and the number of CDS, respectively, with jittered points representing individual genomes. **C** Alluvial plot showing the distribution of host sources and geographic origins of 60 S. agnetis genomes

A high-quality dataset of 60 *S. agnetis* genomes was obtained after rigorous quality filtering (as detailed in Methods), with an average of 55.2 ± 46.3 contigs, 99.3 ± 0.3% completeness, and 1.1 ± 1.0% contamination (Table S1). The genome sizes of these isolates ranged from 2342.0 Kb (strain SNUC_5631: GCA_003040855.1) to 2607.4 Kb (strain BR2804: GCA_023924865.1). The number of protein-coding genes ranged from 2246 in strain SNUC_5631 to 2571 in strain 2044 (GCA_029646385.1). On average, *S. agnetis* members had a genome size of 2469.8 ± 63.6 Kb, 2396.0 ± 72.6 protein-coding genes, 57.7 ± 5.8 tRNA, and 11.2 ± 5.5 rRNA (Fig. 1A and B). The GC content of *S. agnetis* genomes varied slightly (35.7 ± 0.1%). The geographic distribution of the 60 *S. agnetis* strains further demonstrates their widespread occurrence, with isolates originating from various regions, including Australia (*n* = 16, 26.7%), Canada (*n* = 13, 21.7%), the USA (*n* = 11, 18.3%), and countries from other continents (Fig. 1C and Table S1). The host sources were bovine (*n* = 44, 73.3%), chicken (*Gallus gallus*) (*n* = 6, 10.0%), and camel (*n* = 6, 10.0%), alongside sporadic cases from buffalo flies (*n* = 3, 5.0%) and unspecified sources (*n* = 1, 1.7%) (Fig. 1C). The reported isolation sites varied widely and included skin lesions, bovine-associated samples (as defined in the metadata), milk, nasal swabs, homogenized buffalo flies, and bone tissues from lame poultry (Table S1). It should be noted that isolation-source annotations were derived directly from publicly available database metadata (GenBank records), which may be incomplete or heterogeneous across submissions. This distribution indicates that *S. agnetis* isolates have been recovered from multiple animal hosts and geographic regions. Although the majority of genomes were derived from bovine sources, isolates from chickens, camels, and buffalo flies have also been reported, consistent with previous studies describing its association with bovine mastitis and avian lameness [14, 19]. Notably, three isolates derived from homogenized buffalo flies suggest potential arthropod-mediated transmission, aligning with recent evidence implicating this vector in bovine dermatopathology [53]. Going forward, expanding genomic sampling from currently underrepresented hosts (e.g., poultry and camels in this dataset) may facilitate more balanced comparative analyses between bovine and non-bovine isolates, particularly with respect to MGEs, AMR genes, and host-associated virulence factors.

### Phylogenomic analysis of *S. agnetis*

**Fig. 2:**
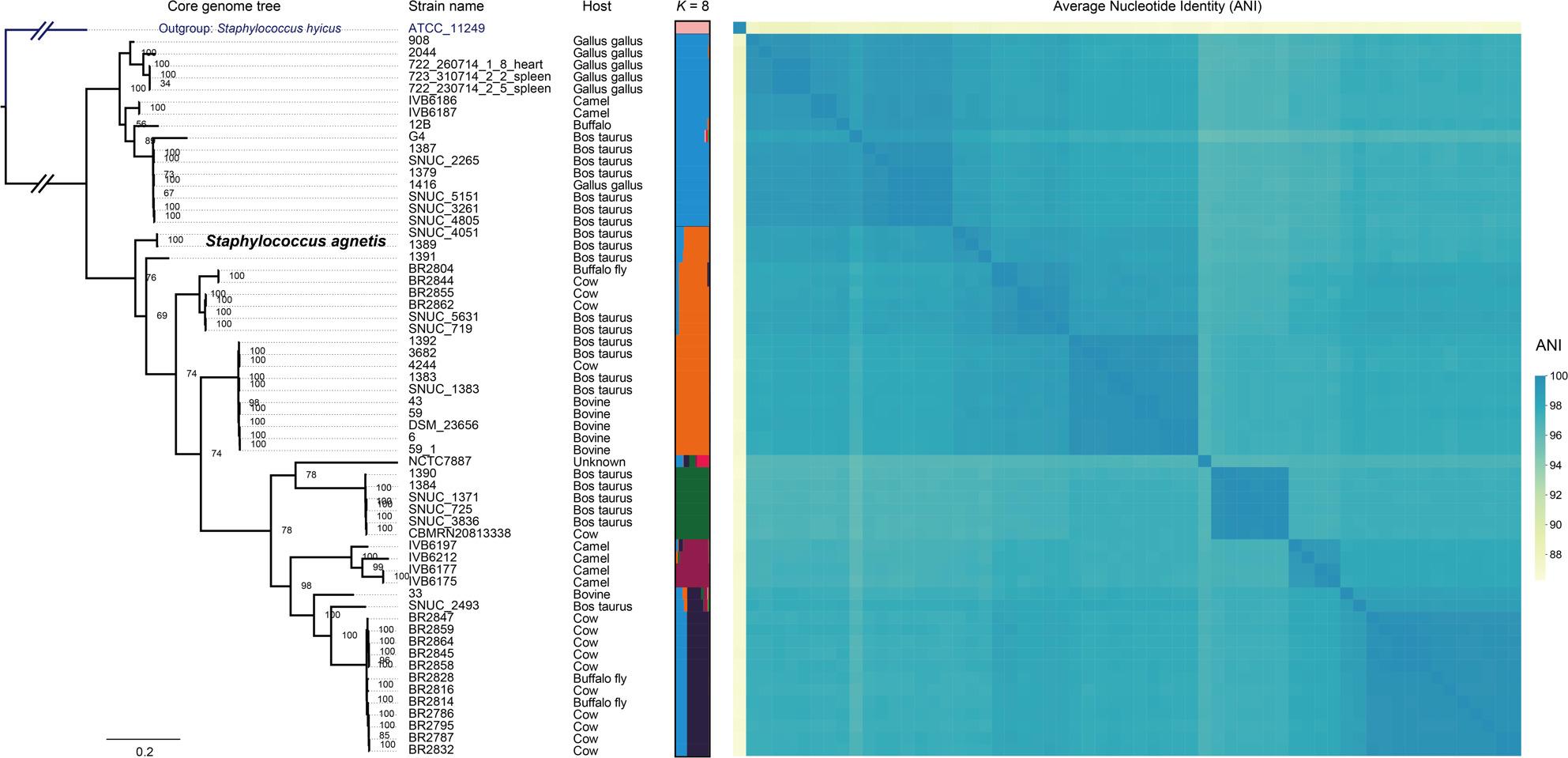
Core genome phylogeny. The maximum likelihood (ML) tree was constructed based on the core gene alignment shared by 60 S. agnetis genomes and one S. hyicus genome (strain ATCC 11249: GCA_000816085.1) as outgroup. The values of the nodes of the tree are the bootstrap values (100 replicates). Adjacent to the tree, colored blocks represent the genetic clusters inferred via STRUCTURE, whereas the accompanying heatmap displays pairwise average nucleotide identities (ANI)

An ML tree was constructed based on the core gene alignment of 60 *S. agnetis* genomes, along with one *S. hyicus* genome (ATCC 11249: GCA_000816085.1) used as an outgroup. In the core genome tree, all *S. agnetis* members formed a monophyletic clade with a long branch length, neighboring the outgroup branch. This suggests that evolutionary divergence occurred between *S. agnetis* and the other *Staphylococcus* species (Fig. 2). The *S. agnetis* clade comprised multiple deep subclades, the members of which exhibit clonality and have very short branch lengths.

The *S. agnetis* members fell into seven intraspecific genetic populations (*K* = 7) based on the STRUCTURE analysis (Fig. 2). Whole-genome comparisons were performed by calculating the ANI for each pair of genomes. The ANI values from pairwise comparisons between *S. agnetis* and *S. hyicus* (ATCC 11249) averaged 87.3 ± 1.7%, indicating a reliable delineation of the genetic relationship between *S. agnetis* and the *S. hyicus* outgroup (Fig. 2). Intraspecific ANI values within *S. agnetis* were greater than 95.8%, averaging 98.0 ± 0.9%. For comparative purposes, we included additional phylogenetic and ANI-based analyses using alternative approaches (Figure S1). The topology of the tree and the genetic relationships were consistent with those of the core-genome alignment-based tree (Fig. 2), showing a similar clustering of the isolates into the same subclades. The ANI values from pyani v0.3.0-alpha also confirmed high intraspecific similarity (≥ 96.4%). This consistency across methods reinforces the robustness of our evolutionary inferences and the monophyletic nature of *S. agnetis*.

### Pan-genome architecture of *S. agnetis*

**Fig. 3:**
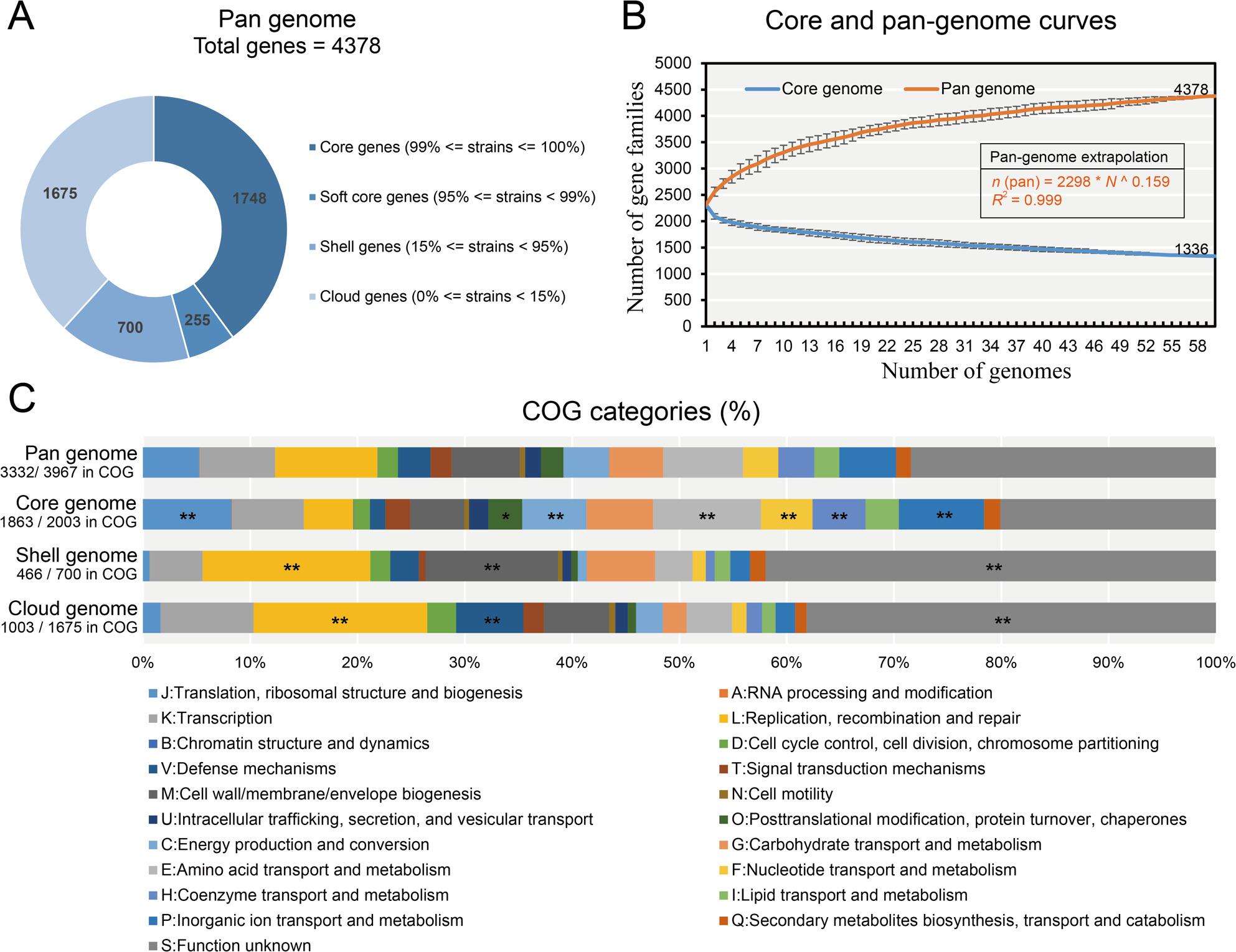
Pan-genome analysis of S. agnetis. **A** Proportional distribution of pan-genome components. The pie chart depicts the percentage compositions of the core, soft core, shell, and cloud gene families within the pan-genome. **B** The cumulative curves for the core and pan-genome of S. agnetis. The curves showed the downward trend of the core gene families and the upward trend of the pan-gene families with the increase in the number of genomes. The X-axis represents the number of genomes, while the Y-axis represents the number of gene families. **C** Distribution of COG categories for pan-genome, core, shell, and cloud gene families, respectively. * Fisher’s exact test: *P*-value < 0.05; ** Fisher’s exact test: *P*-value < 0.01

Pan-genome analysis is a powerful approach for elucidating potential genetic innovations within bacterial species [54, 55]. Analysis of the *S. agnetis* pan-genome revealed that it comprises 4378 homologous gene families, including 1748 core, 255 soft-core, 700 shell, and 1675 cloud gene families (Fig. 3A and Table S2). As shown in Fig. 3A, core genes represent the largest single category (39.9%, 1748 out of 4378), while the accessory genome (shell and cloud) collectively accounts for 54.2%, contributing substantially to genetic diversity. The pan-genome size increased continuously with the sequential addition of genomes, without reaching a plateau within the current dataset of 60 isolates (Fig. 3B). This ongoing expansion, as described by Heaps’ law (*n* = $\:{кN}^{\gamma\:};\:$γ = 0.159; γ > 0), supports an open pan-genome structure in *S. agnetis*, a pattern consistent with observations in other bacterial species, including *Streptococcus agalactiae*, which was the first species analyzed using pan-genome modeling [56]. The absence of a plateau from the genomes analyzed in this study implies that the full genetic diversity of *S. agnetis* has not yet been fully captured, and that additional sequencing may further refine the pan-genome size. Cloud gene families account for the majority (70.5%, 1675 out of 2375) of non-core components. Cloud gene families have been widely reported to be associated with HGT and are thought to contribute to functions such as niche adaptation, antibiotic resistance, and pathogenicity in bacterial species [56, 57].

To gain a better understanding of how each component functions within the pan-genome, we performed a COG analysis to categorize the functions of pan-gene families. However, only 76.1% (3332 out of 4378) of gene families could be assigned to the 22 COG functional categories, since a significant proportion of shell (33.4%, 234 out of 700) and cloud (40.1%, 672 out of 1675) gene families could not be allocated provisional functions (Fig. 3C). The functional divergence observed between core, shell, and cloud components of the pan-genome may reflect the evolutionary dynamics of *S. agnetis* genetic properties. Core gene families were significantly enriched in “J: Translation, ribosomal structure and biogenesis” (Fisher’s exact test: *P-*value < 0.01), “O: Posttranslational modification, protein turnover, chaperones” (Fisher’s exact test: *P-*value < 0.05), “C: Energy production and conversion” (Fisher’s exact test: *P-*value < 0.01), and “Transport and metabolism of amino acid, nucleotide, coenzyme, and inorganic ion” (COG-E, -F, -H, and -P; Fisher’s exact test: *P-*value < 0.01). This core repertoire plays a fundamental role in maintaining basic cellular functions through efficient nutrients (such as amino acids, nucleotides, coenzymes, and inorganic ions) acquisition from ecological niches.

Shell gene families were also enriched in “M: Cell wall/membrane/envelope biogenesis” (Fisher’s exact test: *P-*value < 0.01), suggesting modification of cellular barriers to counteract external disturbances from ecological niches. Both shell and cloud gene families were significantly enriched in “L: Replication, recombination and repair” and “S: Function unknown” (Fisher’s exact test: *P-*value < 0.01), highlighting the significant role of recombination in the genetic innovation of pan-genome evolution. The cloud gene families were also involved in “V: Defense mechanisms” (Fisher’s exact test: *P-*value < 0.01), including type I restriction modification systems (Table S3). These shell and cloud gene families, as variable components of the pan-genome, are closely associated with defense systems and the mobilome, and further investigation may help elucidate the evolutionary dynamics and resistance dissemination in this species.

### The evolutionary scenario of *S. agnetis* characterized by significant genome reduction, gene gain at early-diverging internal nodes, and subsequent gene loss

**Fig. 4:**
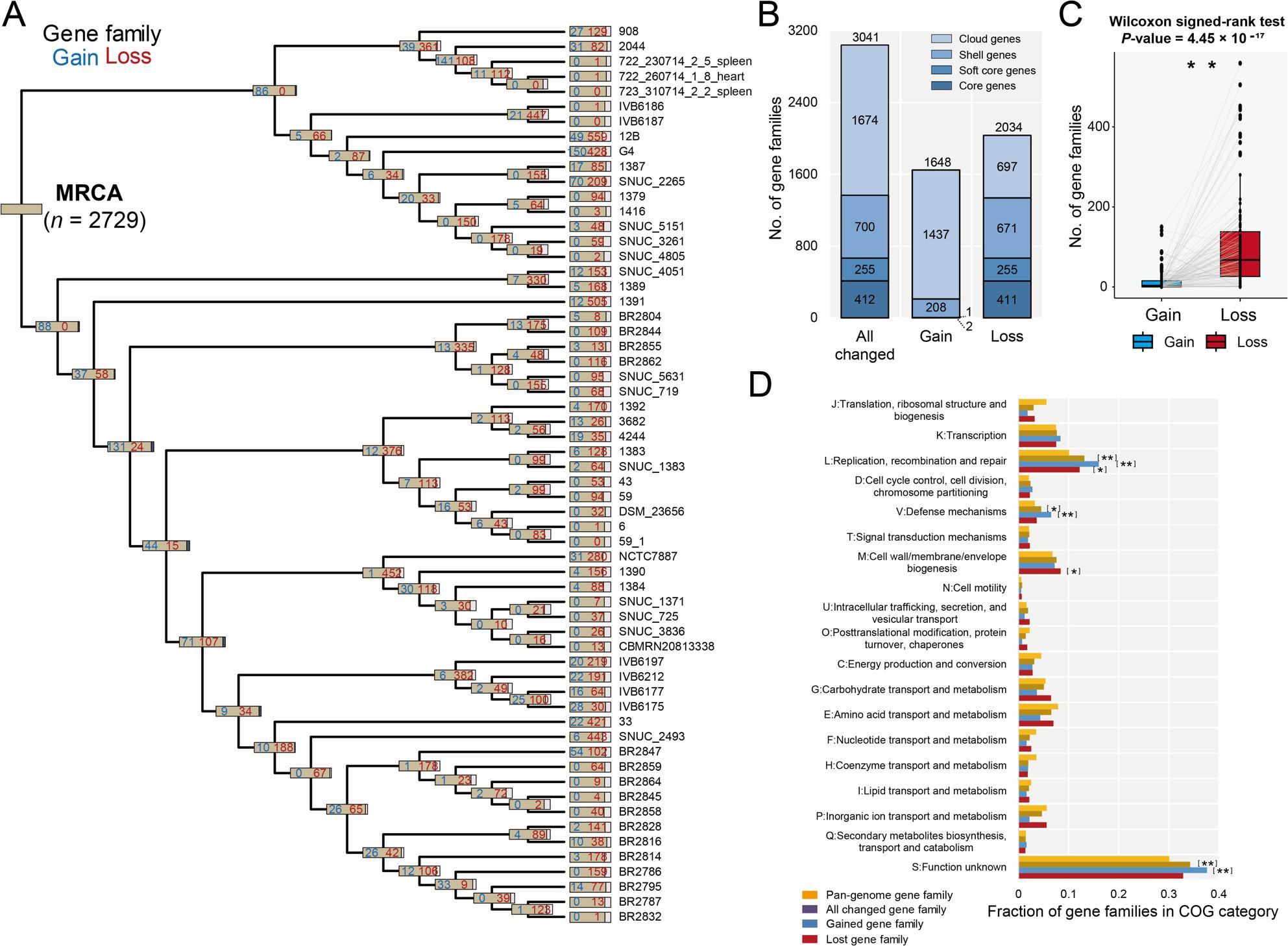
Evolutionary dynamics of gene families within the S. agnetis pan-genome. **A** Gene gain and loss along each evolutionary branch of the core genome tree. Blue and red indicates the numbers of gained and lost gene families for each node, respectively. **B** Bar chart depicting the numbers of core, soft core, shell, and cloud gene families among the changed, gained, and lost gene families, respectively. **C** Box plots comparing gene gains and losses in the S. agnetis pan-genome. The central dark line within the box represents the median. The dots represent the distributions of gene gains (blue) and losses (red) at each node/branch of the core genome tree. The same node/branch is connected by thin black lines between the boxes. ** for Wilcoxon signed-rank test: *P*-value < 0.01. **D** Distribution of COG functional categories for pan, changed, gained, and lost gene families. * Fisher’s exact test: *P*-value < 0.05; ** Fisher’s exact test: *P*-value < 0.01

Gene gain and loss are fundamental to bacterial pan-genome evolution, enabling bacteria to adapt to their environment [31, 58]. To decipher the evolutionary dynamics of the *S. agnetis* pan-genome, we predicted gene gain and loss events by mapping the inferred orthologous gene families onto the core genome tree (Fig. 4A). The most recent common ancestor (MRCA) of *S. agnetis* is inferred to contain 2729 protein-coding genes, which exceeds the average of 2396.0 ± 72.6 genes per genome, indicating significant genome reduction. A total of 3041 gene families, identified as changed/evolving gene families, have undergone gain and/or loss events, comprising 1648 gained and 2034 lost gene families (Fig. 4B). The median rate per node/branch was 3.0 (IQR: 0.0–15.5) gene gains and 67.5 (IQR: 26.0–138.0) gene losses, indicating that gene loss predominated during the evolutionary process of the *S. agnetis* pan-genome compared to gene gain (Wilcoxon signed-rank test: *P*-value < 0.001) (Fig. 4C). However, analysis of key divergence events revealed that a substantial number of gene gain events occurred at early-diverging internal nodes of the phylogeny (Fig. 4A). These gene gain events may have contributed to genomic diversification in *S. agnetis*. However, a substantial proportion of gained gene families belong to the COG-S category (unknown function), limiting functional interpretation. Furthermore, some gained genes are enriched in recombination, DNA repair, and defense-related genes (COG-L and -V; Fisher’s exact test: *P*-value < 0.01) (Fig. 4D). This aligns with the functional enrichment of cloud/shell gene families in COG-L, -V, and -S categories, indicating that gene gain drives the expansion of the pan-genome gene pool of *S. agnetis*.

Conversely, a high rate of gene loss events was observed at the tips of the phylogeny compared with the early/internal nodes, ultimately leading to a reduction in extant individual genomes (Fig. 4A). These numerous instances of gene loss further suggest that the functions of these genes are dispensable. The functional properties of lost gene families were also significantly enriched in COG-L (Fisher’s exact test: *P*-value < 0.05) (Fig. 4D). The concurrent gain and loss of COG-L genes suggests that the recombination machinery acts as both an engine of innovation and a filter of genome reduction in fluctuating environments. Furthermore, gene families related to COG-M (Fisher’s exact test: *P*-value < 0.01) experienced significant losses, resulting in the enrichment of COG-M in the shell genome (Fig. 3C). This reduction in cell wall biogenesis capacity may reflect alterations in cell envelope structure, which could influence interactions with the extracellular environment. Notably, previous studies have shown that cell wall-deficient bacterial forms can acquire extracellular material through engulfment-like processes, although the relevance of such mechanisms in *S. agnetis* remains unclear [59]. Moreover, the loss of cell wall biogenesis genes (COG-M) could potentially drive phenotypic alterations, such as reduced cell wall thickness and enhanced susceptibility to beta-lactam antibiotics, as evidenced by studies on *S. aureus* models where defects in cell wall synthesis pathways led to compensatory changes in wall architecture and resistance profiles [60, 61]. Thus, future work should prioritize experimental validation to decipher the broader implications of this evolutionary trait for antibiotic resistance dynamics.

### Relaxed purifying constraints on shell (cloud) gene families contrast with prevalent positively selected mutations in core (soft) gene families

**Fig. 5:**
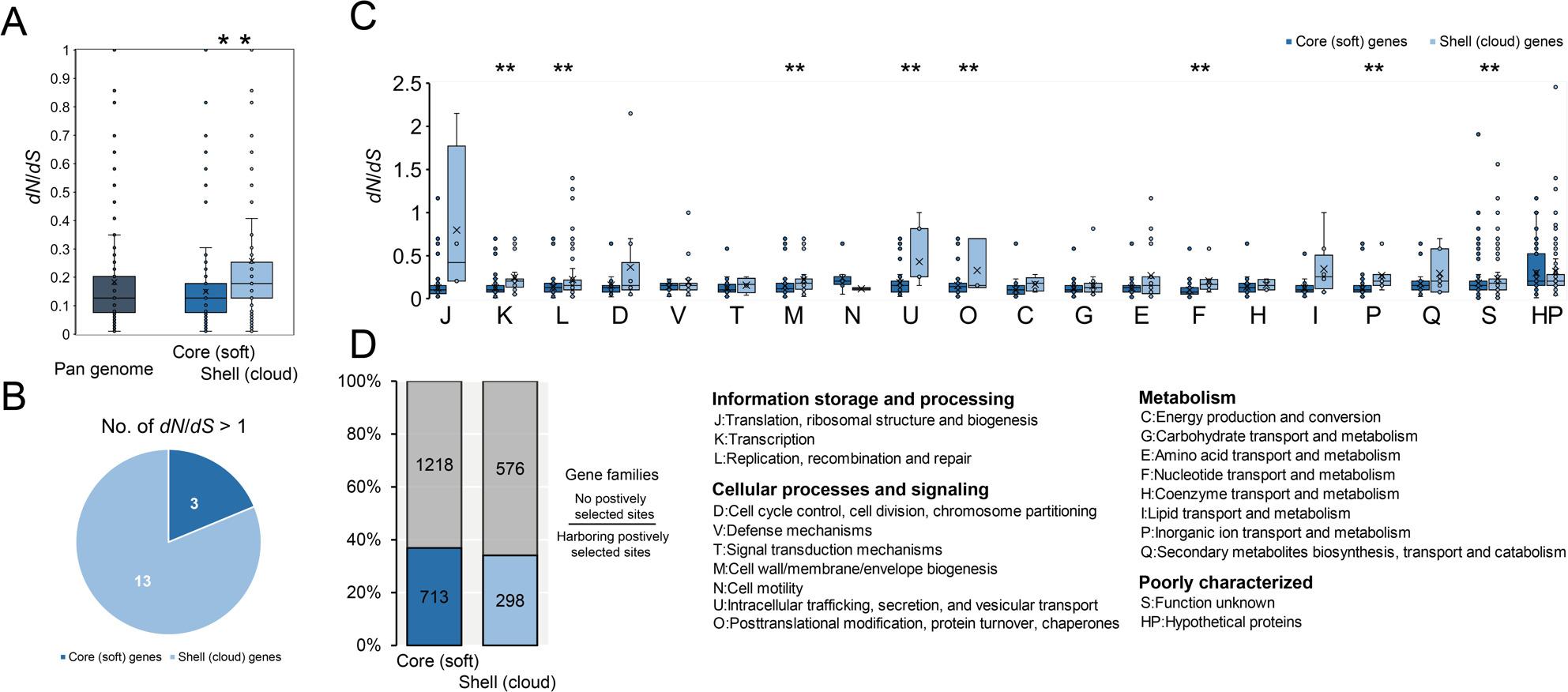
Comparative analysis of selective pressure between different components of the S. agnetis pan-genome. **A** The nonsynonymous/synonymous rate (dN/dS) ratios of pan-gene families and a comparison of gene families between the core (soft) and shell (cloud) genomes. ** t-test: *P*-value < 0.01. **B** Pie chart showing the distribution of positively selected gene families (dN/dS > 1). **C** Comparisons of the dN/dS ratios of gene families in COG functional categories between the core (soft) genome and the shell (cloud) genome. The y-axis range was adjusted to display all data points, including outliers. ** t-test: *P*-value < 0.01. D. A diagram showing the relationship between gene families harboring positively selected sites in the core (soft) and shell (cloud) genomes based on chi-squared proportions tests

Evolutionary dynamics for the adaptation of bacterial species are dominated by natural selection [62]. We calculated the *dN*/*dS* via a codon-level analysis of natural selection across 3284 orthologous families, encompassing 2003 core (soft) and 1281 shell (cloud) gene families present in at least four genomes. Most gene families presented low *dN*/*dS* values, with an average of 0.183 ± 0.190 (Fig. 5A), indicating predominant purifying or stabilizing selection in the *S. agnetis* pan-genome. Bacterial species are generally considered to be subject to a high level of purifying selection, as well as rapid adaptive evolution in changing environments [63, 64]. Of the 16 gene families identified as being under positive selection (*dN*/*dS* > 1), 13 were shell (cloud) gene families and three were core (soft) gene families. The majority of molecular mechanisms for these gene families were recombination (COG-L), translation (COG-J) and unknown (COG-S and HP) (Fig. 5B and Table S4). The level of purifying selection differs between core (soft) and shell (cloud) gene families. Core gene families had an average *dN*/*dS* value of 0.149 ± 0.140, which was significantly lower than that of shell (cloud) gene families (0.258 ± 0.253) (*t*-test: *P*-value < 0.01) (Fig. 5A). These remarkable evolutionary constraints may be due to the functional importance of these core gene families (comprising the ubiquitous backbone of bacterial genomes), maintaining a stable and adapted genomic core [65, 66]. Meanwhile, shell (cloud) gene families (harboring more mobile genes) may be subject to more dynamic evolutionary processes, as reflected by their higher *dN*/*dS* values and enrichment in mobile and accessory functions [67]. Significantly stronger purifying constraints were observed for the core gene families across multiple functional categories, including COG-K, -L, -M, -U, -O, -F, -P, and -S (*t*-test: *P*-value < 0.01) (Fig. 5C). Notably, the COG categories that exhibited differential purifying selection (COG-L, -M, -O, -F, -P and -S) largely coincided with those showing disparities in enrichment between core and shell/cloud components in the pan-genome (Fig. 3C), with the exception of COG-K and -U.

Despite purifying selection acting on the entire coding regions of gene families, we observed numerous codon sites within the pan-genome with significant evidence of positive selection (posterior probability ≥ 0.9). A total of 1011 gene families were found to contain one or more such codon sites (Table S5). Of these, 713 (70.5%) were core (soft) gene families and the remaining 298 (29.5%) were shell (cloud) gene families (Fig. 5D). Notably, despite the entire coding region undergoing stronger purifying selection, core (soft) gene families harbored disproportionately more positively selected sites (36.9%, 713 out of 1931) than shell (cloud) gene families (34.1%, 298 out of 874), though this enrichment did not reach statistical significance (Chi-squared test: χ2 = 1.966, df = 1, *P*-value = 0.161) (Fig. 5D). This finding implies evolutionary modifications at the residue level in conserved proteins.

### AMR profile of *S. agnetis* represented by a low number of AMR genes

Prediction of AMR phenotypes and detection of AMR genes were analyzed using ResFinder v4.3.1 [47]. The AMR phenotypic profile of *S. agnetis* was predicted to be resistant to nine antibiotics in four major classes: aminoglycosides, beta-lactams, fosfomycin, and tetracyclines (Table S6). A total of five AMR genes were identified, including *aadD*, *blaZ*, *fosD*, *tet*(K), and *tet*(L) (Table S7). The *aadD* gene encodes an adenyltransferase aminoglycoside-modifying enzyme (AAD(4’,4’’)) that confers resistance to aminoglycosides such as kanamycin and neomycin [68]. The *blaZ* gene, encoding an extracellular beta-lactamase that inactivates the antibiotic by hydrolyzing beta-lactam ring, was identified in five *S. agnetis* genomes [69]. The *fosD* gene mediating fosfomycin resistance occurs in *S. aureus* [70]. Additional resistance genes, *tet*(K) and *tet*(L), code for resistance to the antibiotic tetracycline.

**Fig. 6:**
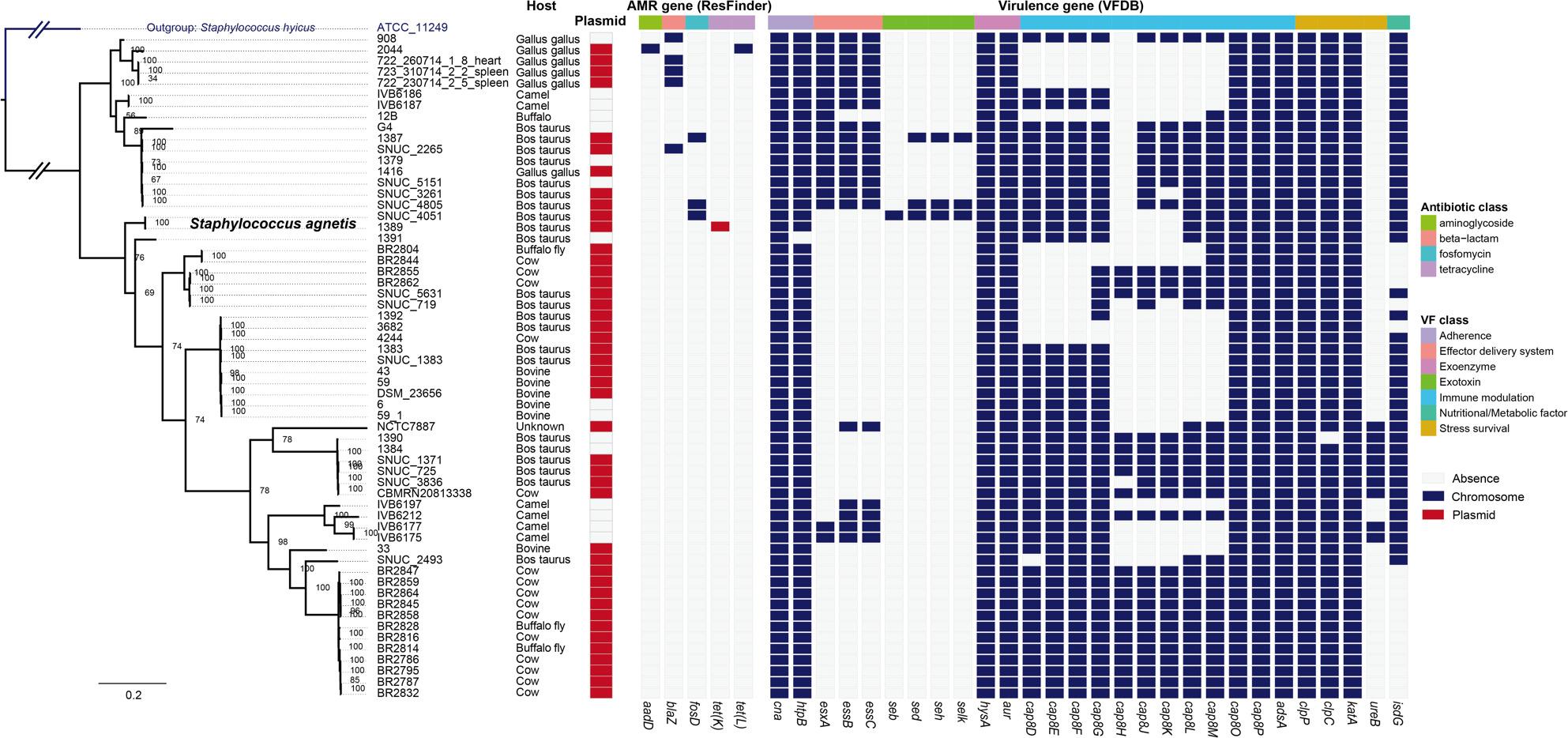
The genotypic profiles of antimicrobial resistance (AMR) genes and putative virulence-related genes across 60 S. agnetis genomes. The genome order corresponds to the core genome tree. Heatmaps depict the distribution of AMR and putative virulence-related genes. Blue coloring represents the presence of a gene, and gray represents absence

Ten (16.7%) *S. agnetis* genomes were found to harbor these AMR genes, with only one genome (strain 2044) containing two AMR genes (*aadD* and *tet*(L)) and the others containing only one AMR gene (Fig. 6). While the ten AMR-gene-harboring genomes were equally distributed between chicken and bovine hosts (five isolates each), their prevalence within each host group differed markedly, being detected in 83.3% (5 out of 6) of chicken isolates and 11.4% (5 out of 44) of bovine isolates (Fisher’s exact test: *P*-value = 0.00065; odds ratio = 39.0, 95% CI 3.76–404.95). However, given the limited number of chicken isolates, this result should be interpreted cautiously and does not by itself support broad conclusions regarding the poultry environment. Previous studies have reported that poultry-associated environments can harbor a wide diversity of bacterial species [71], with non-aureus staphylococci frequently detected and reported to carry AMR genes, posing a potential public health concern [72].

These AMR genes represent cloud gene families, as each individual gene is present in a small fraction of genomes (≤ 15% prevalence, e.g., the most prevalent gene, *blaZ*, is detected in only five out of 60 genomes), consistent with their sporadic distribution and potential association with MGEs (Fig. 6). Further analysis indeed revealed that the *tet*(K) gene in *S. agnetis* 1398 was located in the MGE region (IS*Sau6*-*tet*(K)*-*IS*Sau6*) of the plasmid sequence detected (contig: WMFK01000034.1) (Figure S2). A BLASTn search of the NCBI Non-Redundant (NR) database was performed using the full sequence of the *tet*(K)-harboring contig. Interestingly, ten highly similar sequences (with 99% query coverage and > 99% nucleotide identity) were identified. These were all chromosomes of *Staphylococcus pseudintermedius* strains (Table S8), suggesting possible genetic exchange between plasmid and chromosomal elements. IS-mediated AMR transmission (including between plasmids and chromosomes) has been reported to play a role in the development of antibiotic resistance in *S. aureus* strains [73, 74]. Although there are only a few AMR genes, plasmid nucleotide sequences were detected in the majority (73.3%, 44 out of 60) of the *S. agnetis* genomes (Fig. 6 and Table S9), suggesting a high prevalence of plasmid-associated sequences. Therefore, the acquisition of plasmid-driven AMR genes cannot be ignored in the emergence of antibiotic resistance in *S. agnetis*. Meanwhile, the transmission capacity of the plasmid remains subject to experimental verification (e.g., through experiments investigating conjugative transfer efficiency).

### Potential virulence-related genotypic profile in the *S. agnetis* pan-genome

Several pathogenic phenotypes of *S. agnetis* have been reported [17]. To evaluate the potential pathogenicity of *S. agnetis*, we identified 28 putative virulence-related genes involved in adherence, effector delivery system, exotoxins, exoenzymes, immune modulation, stress survival and nutritional factors. These genes were identified based on sequence homology to entries in the VFDB database. Given the absence of *S. agnetis*-specific annotations in VFDB, these predictions rely on similarity to virulence factors characterized in related species, primarily *S. aureus*. Therefore, the identified genes should be interpreted as candidate virulence-related homologs rather than experimentally validated virulence determinants. Functional interpretations were based on genes with consistent annotations and biologically plausible roles, and the inferred virulence potential should be considered preliminary and requires experimental validation.

All genes are located on chromosomes, and most (82.1%, 23 out of 28) genes revealed significant homology with *S. aureus* (Table S10). The potential for adherence was represented by two core genome components, the *cna* and *htpB* genes. The *cna* gene encodes a *S. aureus* collagen adhesin that mediates bacterial adherence to collagen substrates and tissues that are strongly associated with the pathogenesis of osteomyelitis and septic arthritis [75, 76]. The type VII secretion system (T7SS) in *S. aureus* has been reported to play a key role in promoting EsxA-mediated disruption of the host cell membrane, intracellular survival, long-term persistence, and competitiveness [77, 78]. We identified an incomplete T7SS in approximately 20 *S. agnetis* genomes, comprising the shell gene families *esxA* (encoding a secretory protein) and *essB*/essC (encoding membrane-associated proteins). The functional role of this incomplete T7SS in *S. agnetis* warrants further investigation. Three genomes contained multiple homologs of the SE encoding genes. Strain SNUC_4051 was found to harbor homologs of *seb*, *sed*, *seh*, and *selk*, while SNUC_4805 and 1387 both contained *sed*, *seh*, and *selk* homologs. The sporadic distribution of these SE homologs (cloud genes) may be consistent with possible horizontal acquisition, aligning with the established localization of SE genes on MGEs (e.g., SaPIs, prophages) in *S. aureus* [79]. SEs are potent virulence factors that are typically associated with *S. aureus* pathogenesis and have been shown to be responsible for a range of symptoms including emesis, immune activation, and toxic shock syndrome [79]. Consequently, these sporadically distributed SE homologs in *S. agnetis* necessitate functional characterization to evaluate potential biological activities and pathogenicity.

Regarding “Immune modulation”, the *cap8* operon, encoding enzymes essential for type 8 capsule biogenesis, was identified as a variable virulence trait within the *S. agnetis* pan-genome. Excluding the core genes *cap8O* and *cap8P*, the remaining components, classified as shell genes, exhibited heterogeneous distribution across the 60 genomes (Fig. 6). Notably, components such as *cap8H*, *cap8J*, and *cap8K* displayed fragmented occurrence, with *cap8H* absent in 65.0% (39 out of 60) genomes. It has also been observed in *S. aureus* capsule-deficient strains from osteomyelitis patients, where the loss of cap gene has been shown to correlate with chronic persistence through impaired phagocytosis resistance [80, 81]. The *adsA* gene, encoding an adenosine synthase that converts nucleotides into immunosuppressive adenosine, likely dampens host immune responses to enable bacterial evasion [82]. Additional virulence-related genes were associated with stress survival (*clpC*, *clpP*, *katA*, and *ureB*) and nutritional/metabolic factors (*isdG*).

## Conclusion

This study provides the first comprehensive pan-genome analysis of *S. agnetis*, delineating its genetic diversity, evolutionary dynamics, and potential pathogenic features. The open pan-genome architecture of *S. agnetis*, dominated by cloud gene families, is consistent with ongoing gene flux and genomic plasticity. This pattern is further supported by the enrichment of COG-L and -V categories among gained and lost gene families. Evolutionary reconstruction indicates that genome reduction from the MRCA occurred predominantly through gene loss, particularly affecting COG-M. This pattern may reflect changes in cell envelope structure, which could influence interactions with the extracellular environment. Although only a limited number of AMR genes were identified, the emergence of plasmid-mediated AMR determinants and the prevalent carriage of plasmid sequences in the majority of genomes underscore the potential for resistance dissemination. The identified putative virulence-related genes, largely homologous to those characterized in *S. aureus*, together with the sporadic distribution of enterotoxin gene homologs, suggest a potential for pathogenesis and immune interaction in *S. agnetis*. These findings collectively highlight that *S. agnetis* may represent an evolving pathogen with putative virulence potential and emerging AMR traits, warranting continued surveillance of this species in animal-associated settings and further monitoring of resistance development.

## Supplementary Information

Supplementary Material 1: Figure S1. The maximum likelihood (ML) tree was constructed based on core-genome single-nucleotide polymorphisms (SNPs) shared by 60 S. agnetis genomes, using Parsnp v2.1.1 and FastTree v2.1.10. The accompanying heatmap, located adjacent to the tree, displays the average nucleotide identities (ANI) calculated by pyani v0.3.0-alpha using the ANIm model

Supplementary Material 2: Figure S2. Expanded view of the MGE region containing two ISSau6 elements and the AMR gene tet(K)

Supplementary Material 3: Table S1. The public genome sequences (n = 60) utilized in this study were obtained from the NCBI GenBank and EzBioCloud Databases

Supplementary Material 4: Table S2. List of the pan-gene families found in 60 S. agnetis genomes, categorized as core, soft core, shell or cloud

Supplementary Material 5: Table S3. Annotation results of cloud gene families enriched in COG category V (Defense mechanisms) using eggNOG-mapper v2.1.9

Supplementary Material 6: Table S4. List of the positively selected gene families (dN/dS > 1)

Supplementary Material 7: Table S5. List of gene families with positively selected sites

Supplementary Material 8: Table S6. Antimicrobial susceptibility profiles of Staphylococcus agnetis by AMR phenotype prediction using ResFinder v4.3.1 with a cutoff at 80% nucleotide identity and 60% nucleotide coverage. N, no resistance; R, resistant

Supplementary Material 9: Table S7. List of identified antimicrobial resistance (AMR) genes in the Staphylococcus agnetis genomes (ResFinder v4.3.1)

Supplementary Material 10: Table S8. List of homologous sequences that are highly similar to the tet(K)-harboring contig (strain 1398: WMFK01000034.1), as identified in the NCBI Non-Redundant (NR) database

Supplementary Material 11: Table S9. List of identified plasmid sequences within the Staphylococcus agnetis species complex genomes (assembly type: contig or scaffold) in this study

Supplementary Material 12: Table S10. List of putative virulence-related genes identified in the Staphylococcus agnetis genomes (VFDB database)

## Data Availability

The genomes that were used are listed in Table S1 (available with the online version of this article) and the data are publicly available through the National Center for Biotechnology Information (NCBI) GenBank database.

## Abbreviations

AMR: Antimicrobial resistance
ML: Maximum-likelihood
SNP: Single-nucleotide polymorphism
dN/dS: Ratios of nonsynonymous to synonymous substitution rates
ICE: Integrative and conjugative element
ANI: Average nucleotide identity
COG: Clusters of Orthologous Groups
HGT: Horizontal gene transfer
VFDB: Virulence Factors Database
MRCA: Most recent common ancestor
MGE: Mobile genetic elements
SE: Staphylococcal enterotoxin
T7SS: Type VII secretion system
